# Altered MAIT cell activation characteristics with impaired IL-10 and IL-17A response programs in active Crohn’s disease

**DOI:** 10.1093/jimmun/vkag271

**Published:** 2026-10-26

**Authors:** Elli Mouchtaridi, Jonas Halfvarson, Jeffrey Y W Mak, David P Fairlie, Jenny Mjösberg, Peter Bergman, Johan K Sandberg, Caroline Boulouis

**Affiliations:** Center for Infectious Medicine, Department of Medicine, Karolinska Institutet, Stockholm, Sweden; Department of Gastroenterology, Faculty of Medicine and Health, Örebro University, Örebro, Sweden; Centre for Chemistry and Drug Discovery, Australian Research Council Centre of Excellence for Innovations in Peptide and Protein Science, Institute for Molecular Bioscience, The University of Queensland, Brisbane, Australia; Centre for Chemistry and Drug Discovery, Australian Research Council Centre of Excellence for Innovations in Peptide and Protein Science, Institute for Molecular Bioscience, The University of Queensland, Brisbane, Australia; Center for Infectious Medicine, Department of Medicine, Karolinska Institutet, Stockholm, Sweden; Clinical Lung and Allergy Research Unit, Medical Unit for Lung and Allergy Diseases, Karolinska University Hospital Huddinge, Stockholm, Sweden; Department of Laboratory Medicine, Division of Clinical Immunology, Karolinska Institutet, Stockholm, Sweden; Department of Clinical Immunology and Transfusion Medicine, Karolinska University Hospital, Stockholm, Sweden; Center for Infectious Medicine, Department of Medicine, Karolinska Institutet, Stockholm, Sweden; Center for Infectious Medicine, Department of Medicine, Karolinska Institutet, Stockholm, Sweden

**Keywords:** Crohn’s disease, human, interleukins, intestinal tissue, MAIT cells

## Abstract

Mucosa-associated invariant T (MAIT) cells are innate-like MR1-restricted T cells broadly distributed in human tissues. MAIT cells adapt their antigen-specific response patterns depending on cytokine cues such as IL-12 or IL-18 in the microenvironment, shifting toward either IL-10^+^ or IL-17^+^ profiles. However, if such patterns are maintained in the context of inflammatory diseases is unknown. Here, we investigated MAIT cell functional plasticity in Crohn’s disease (CD). Circulating MAIT cells in CD patients were impaired both in terms of numbers, and in terms of interferon γ (IFN-γ) and tumor necrosis factor (TNF) expression, and cytotoxic properties in response to MR1-presented antigen. However, the MAIT cell regulatory IL-10^+^ profile and the IL-17A^+^ profile were compromised only during active disease and relatively preserved during remission. Transcriptional analysis of intestinal T cells suggested that MAIT cells in CD displayed elevated *GNLY* and *NKG7* expression, while transcripts associated with regulation and quiescence characterized MAIT cells in healthy donor control tissue. There were also distinct transcriptional signatures according to localization, where intra-epithelial MAIT cells exhibited a tissue resident regulatory profile, whereas those in the lamina propria showed signs of activation and recruitment. Finally, we assessed whether treatments for CD influenced MAIT cell functionality *in vitro*. Corticosteroids and upadacitinib inhibited MAIT cell activation, yet upadacitinib promoted increased GM-CSF and IL-17A expression in the context of combined antigen and IL-18 stimulation. These findings indicate that MAIT cell functional response patterns are influenced by CD disease stage, treatment, and tissue localization.

## Introduction

Mucosa-associated invariant T (MAIT) cells are unconventional T cells with antimicrobial properties.^1^ Through their semi-invariant T cell receptor (TCR), MAIT cells recognize metabolites derived from the vitamin B2 pathway, expressed by many species of bacteria and yeast.^2–4^ The expression of cytokine receptors such as IL-12R, IL-15R, and type I interferon receptors by MAIT cells allows activation and co-activation by cytokines.^1^ In response to combined TCR and cytokine activation, MAIT cell display a broad activation profile with the release of interferon γ (IFN-γ), tumor necrosis factor (TNF), IL-17A and cytotoxic proteins, such as granzyme B (GzmB). Furthermore, MAIT cells adapt their functional effector response during antigen recognition to the type of cytokine cues present in the microenvironment. MAIT cell activation with MR1-presented 5-(2-oxopropylideneamino)-6-D-ribitylaminouracil (5-OP-RU) antigen in combination with IL-12 drives MAIT cells towards a partially regulatory state, characterized by IL-10 secretion and expression of inhibitory receptors.^5^ In contrast, MAIT cell activation with 5-OP-RU and IL-18 shifts MAIT cells toward a pro-inflammatory state, marked by elevated levels of IL-17A, IFN-γ and TNF.^5^

MAIT cells are present at barrier sites such as the lungs, liver, and intestines and comprise up to 10% of T cells in blood.^6^^,^^7^ Resident MAIT cells in the gastrointestinal tract express high levels of the immunomodulatory molecule CD39, suggesting a potential regulatory role at this site.^6^ MAIT cells serve as a first line of defense against infection and play a protective role in some bacterial and viral infections.^8^ MAIT cells have also been shown to participate in tissue repair, providing accelerated wound healing in skin and the intestinal barrier.^9–11^ Notably, despite their CD39^+^ immunomodulatory character in the intestines, MAIT cells respond quite vigorously to infection with intestinal bacterial pathogens including *Salmonella enterica* and enterotoxigenic *Escherichia coli* (ETEC).^12^^,^^13^ In inflammatory diseases, emerging data suggest that MAIT cells can play both pathogenic and protective roles.^8^ Thus, the role of MAIT cells in inflammatory disease is still controversial and few studies have addressed their role in inflammatory bowel disease (IBD).

IBD includes 2 different diseases: ulcerative colitis and Crohn’s disease (CD). In this study, we focused on CD, which is characterized by chronic inflammation in several parts of the gastrointestinal tract. CD alternates between episodes of active inflammation and periods of remission.^14^ Over time, the disease can become progressive and lead to serious intestinal and extraintestinal complications, such as strictures, cholangitis, or colorectal cancer, which may require hospitalization and surgery.^14^ Most of the complications are the result of immune dysfunction.^14^ The cause of CD is unknown; dysregulated immune responses, altered microbiota as well as genetic and environmental factors are involved in its etiology and pathogenesis. Polymorphisms in the *IL10*, *IL10RA* and *IL10RB* genes are associated with very early onset IBD, including CD.^15^^,^^16^ Given that MAIT cells can express high amounts of IL-10,^5^ it is important to understand their involvement in IBD pathogenesis. Previous studies have shown that MAIT cells are reduced in the circulation in IBD patients,^17–20^ and that this loss correlates with disease activity.^20^ Interestingly, MAIT cell reduction in the circulation is accompanied by a concomitant accumulation at lesion sites,^17^^,^^19^ suggesting possible recruitment from the periphery. In addition, plasma levels of MAIT cell agonist are elevated during the active stage of IBD,^21^ which may contribute to MAIT cell activation and recruitment to inflamed tissues. Upon PMA/ionomycin stimulation, blood MAIT cells from CD patients produced more IL-17A than healthy controls,^17^^,^^20^ suggesting that these cells might be skewed toward IL-17A production in IBD. Finally, recent studies in mouse models of colitis found that colonic MAIT cells display a Th17 phenotype,^22^ and contribute to disease severity,^22^^,^^23^ while results by El Morr et al. rather indicated that intestinal MAIT cells contribute to tissue repair during colitis.^10^

In this study, we investigated how MAIT cells from CD patients respond to cues in the microenvironment upon antigen recognition and whether the regulatory abilities of MAIT cells are maintained in CD. We discovered that certain MAIT cell functions are impaired in CD, whereas MAIT cell production of IL-10 and IL-17A show divergent patterns and are relatively preserved in remission and reduced during active CD. Leveraging single cell RNA-sequencing (scRNAseq) data from intestinal T cells, we found that MAIT cells have enhanced levels of transcripts for granulysin and NKG7 in CD, and that the MAIT cells transcriptional profile differs between the lamina propria and the epithelium. Finally, we demonstrated that corticosteroids and the JAK kinase inhibitor upadacitinib, which are routinely used in the treatment of CD, inhibited MAIT cell activation, while 5-ASA and thiopurine had milder effects.

## Materials and methods

### Ethics statement

Written informed consent was obtained from all donors in accordance with the study protocol to the provisions of the Declaration of Helsinki, and ethical approval was obtained from the Regional Ethics Review Board in Stockholm (Dnr 2020-02604) and the Regional Ethics Review Board in Uppsala (Dnr 2010/313).

### Study population

Patients with CD were prospectively included from the population-based IBD cohort of Örebro University Hospital, Sweden.^24^ Disease location and behavior was classified according to the Montreal classification. Clinical disease activity was evaluated using the Harvey-Bradshaw Index (HBI), with remission defined as a HBI <5. A summary of the clinical characteristics of the cohort is shown in Table 1. At inclusion, peripheral blood samples were obtained from the patients with CD patients and from healthy controls, ie blood donors without any history of chronic gastrointestinal disease, at Örebro University Hospital, Sweden.

**Table 1.** Demographics and clinical characteristics of patients with Crohn’s disease and healthy controls.

	Healthy controls	CD remission	CD active
*n*	20	19	11
Sex, *n*			
Men	11	10	8
Women	9	9	3
Age, years (median)	63	69	69
Location, *n*			
Ileal (L1)	N/A	7	3
Colonic (L2)	N/A	3	1
Ileocolonic (L3)	N/A	9	7
Behavioral, *n*			
Non-stricturing, non-penetrating (B1)	N/A	7	1
Stricturing (B2)	N/A	11	9
Penetrating (B3)	N/A	1	1
Treatment	none		
5-ASAs		2	3
Corticosteroid		1	2
Thiopurine		1	1
NSAID		–	2

5-ASAs, 5-aminosalicylates, NSAID: non-steroid anti-inflammatory drug.

CD, Crohn’s disease.

### Blood processing

Peripheral blood mononuclear cells (PBMCs) from the IBD cohort were obtained from blood collected in heparin tubes after isolation by Ficoll density gradient separation (Axis shield, AXS-1114544-AXS-1115758) within 1 h. PBMCs were then washed, counted, and frozen in FCS with 10% DMSO and stored at −180 °C. For the drug functional assay, peripheral blood samples were collected from healthy adult volunteers at the Blood Transfusion Clinic at the Karolinska University Hospital Huddinge, Sweden. Fresh PBMCs were obtained through isolation by Ficoll density gradient separation (Cytivia, 17144003).

### Antigens

The 5-OP-RU antigen was synthesized as previously described.^25^

### MAIT cell functional assay

For the samples from CD patients and HC, PBMCs were thawed in complete medium composed of RPMI-1640 (Cytivia, SH30096.FS) supplemented with 2 mM L-Glutamine (Cytivia, SH30034.01), 10% FCS (Sigma, F2442), HEPES (Cytivia, SH30237.01), 50 μg/mL gentamicin (Gibco, 15750060) and 100 μg/mL normocin (Invivogen, ant-nr-5), washed, and rested for 24 h in complete media. Stimulation was performed with 5-OP-RU at 16 nM in the presence of IL-12 at 10 ng/mL (PeproTech, 210-12), or IL-18 at 100 ng/mL (MBL, B001-5) for 24 h. During the last 6 h of culture, monensin and brefeldin A (BD Biosciences, 554724, 555029) were added. Intracellular Vα7.2 TCR staining was performed to identify MAIT cells in the functional assay.

For the drug testing experiments, PBMCs were stained with anti-Vα7.2-PE or PE-Cy7 (3C10, Biolegend) followed by anti-PE microbeads (Miltenyi, 130-048-801) and passed through a LS column (Miltenyi, 130-042-401) as per manufacturer’s instructions to isolate Vα7.2^+^ cells.^26^ After isolation, Vα7.2^+^ cells were rested for 48 h at 37 °C, 5% CO_2_ in complete medium. Stimulation was performed using THP-1-pulsed 5-OP-RU at 16 nM for 3 h, before adding Vα7.2^+^ cells at a 2:1 ratio for 24 h in the presence of the cytokines IL-12, at 10 ng/mL (PeproTech, 210-12), or IL-18 at 100 ng/mL (MBL, B001-5). During the last 6 h of culture, monensin and brefeldin A (BD Biosciences, 554724, 555029) were added. Drugs or DMSO (Sigma, D2650) control were added at the indicated concentration for the entire co-culture.

### Drug preparation

Upadacitinib (Abbvie), prednisolone (Merck, P6004), azathioprine (Merck, A4638), and mesalazine (Merck, Y0000297) in powder form were resuspended in DMSO at concentrations of 200 mM.

### Flow cytometry

Tetramer staining with 5-OP-RU-hMR1 was performed at room temperature for 40 min before proceeding with extracellular staining. Intracellular staining was performed with Fixation/Permeabilization (BD Biosciences, 554714). The Vα7.2 staining was performed both extracellular and intracellular to catch TCR downregulation. Reagents used in flow cytometry are listed in Table S1. Samples were acquired on a BD Symphony A3 (BD Biosciences) and analyzed using FlowJo software v.10.10 (TreeStar). Polyfunctionality analysis was performed using SPICE software v6.1.^27^

### scRNAseq analysis

Count matrices were downloaded from GEO accession number GSE157477,^28^ and processed with Scanpy (1.10.3). Cells with less than 600 genes, less than 1,000 counts, with more than 15% mitochondrial reads and less than 15% ribosomal reads were filtered out. Genes detected in less than 3 cells, *MALAT1* and hemoglobin genes were filtered out. Using scrublet (0.2.3), doublets were removed. Count was normalized to 10 000 and log-transformed before selection of highly variable genes with a min_mean of 0.001 and max_mean of 10. Principal component analysis was used for dimensionality reduction. Batch effect between donors was removed with Harmony (v.0.0.6). Finally, Leiden clustering was performed at a resolution of 0.5. MAIT cells were identified with the co-expression of *SLC4A10*, *KLRB1*, *IL7R,* and *CD8A* expression > 1. Differential gene expression (DGE) was performed with Scanpy on non-batch corrected data using Wilcoxon rank-sum test with Benjamini-Hochberg correction. MAIT cell IL-10 and inflammatory signatures were defined by transcripts upregulated,^5^ respectively, in *IL10*^+^ cells in 5-OP-RU, 5-OP-RU plus IL-12 and 5-OP-RU plus IL-23 versus *IL10*- counterpart cells with *P*-value <0.05 and log fold change > 0.6, and genes upregulated in 5-OP-RU plus IL18 versus 5-OP-RU with *P*-value <0.05 and log fold change > 0.8 (Table S2).

For verification of the MAIT cell identification strategy in scRNAseq data we used previously published data on MAIT cells isolated by flow cytometry sorting.^6^ Processed data were downloaded from GEO accession number GSE247338 in rds format. The data were processed with Scanpy (1.10.3) after conversion to anndata. The data set included precomputed UMAP embeddings and annotations generated in the original Seurat analysis. The UMAP embeddings and cell annotations were retained and used for visualization of cell population. MAIT cells were identified with the co-expression of *SLC4A10*, *KLRB1*, *IL7R*, and *CD8A* and expression > 1.

### Statistical analysis

Statistical analyses were performed using Prism 10.5.0 using non-parametric tests. The Wilcoxon test or Friedman test followed by Dunn’s multiple comparison were used to compare non-normally distributed paired data. For unpaired data, the Mann–Whitney test or Kruskal–Wallis test, followed by Dunn’s multiple comparisons were performed.

## Results

### MAIT cells are functionally impaired in Crohn’s disease but still respond to cytokine cues in the microenvironment

We previously found that MAIT cell responses to antigenic stimulation are shifted by the cytokine microenvironment, with IL-12 driving MAIT cells toward a profile including IL-10 expression, whereas IL-18 biases MAIT cells toward a highly activated inflammatory cytokine profile including IL-17A.^5^ To assess if these distinctive MAIT cell functional response profiles were maintained in CD, PBMCs from healthy controls (HC) and CD patients in a remission state or with active disease (Table 1) were stimulated with MAIT cell antigen 5-OP-RU alone, or in combination with IL-12 or IL-18, or with a combination of IL-12 and IL-18. Responses and cytokine expression were measured by intracellular flow cytometry staining. Circulating MAIT cells in patients with CD were reduced, consistent with previous studies (Fig. 1A and B, Fig. S1A).^17^^,^^20^ Expression of IL-10 was induced following 5-OP-RU plus IL-12 stimulation, and this pattern was maintained in CD patients (Fig. 1C and D, Fig. S1B). Stimulation with 5-OP-RU plus IL-18, or with 5-OP-RU plus IL-18 combined with IL-12, shifted MAIT cells toward the production of IL-17A, IFN-γ, and TNF in both HC and CD patients (Fig. 1E-G). IFN-γ and TNF were induced at intermediate levels by 5-OP-RU and IL-12 stimulation, and this pattern was sustained in disease states (Fig. 1F and G). GzmB expression and CD107a degranulation by MAIT cells were induced by either mode of stimulation in both HC and CD patients (Fig. S1C and D).

**Figure 1:**
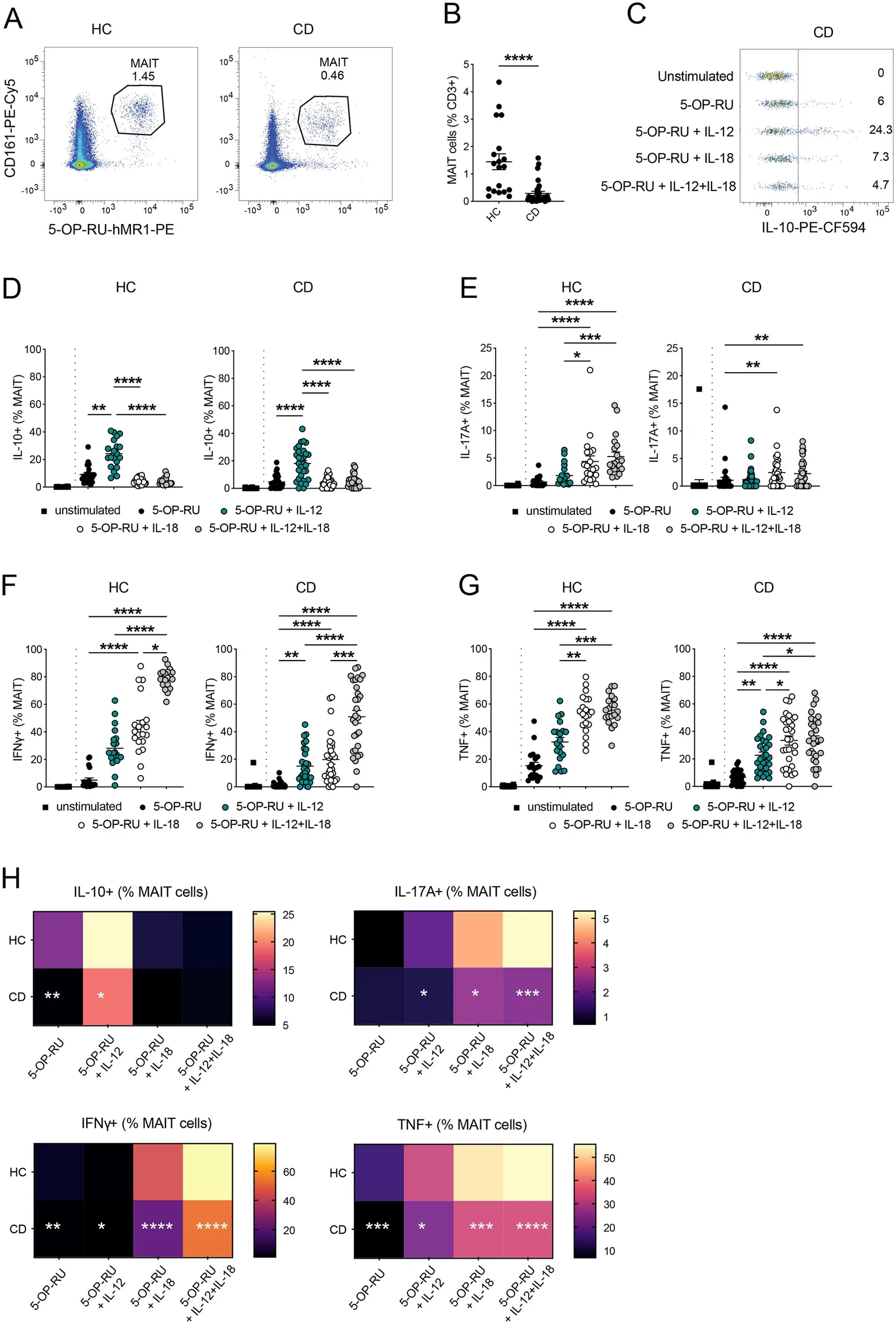
Peripheral blood MAIT cell cytokine expression profile in Crohn’s disease patients is shifted by the cytokine environment. Representative FACS plots (A) and summary data (B) of the percentage of circulating MAIT cells (5-OP-RU-hMR1^+^CD161^+^) in healthy controls (HC), and Crohn’s disease (CD). (C) Representative FACS plots of IL-10 expression by MAIT cells in CD at the indicated stimulation. Summary data of percentage of MAIT cells expressing IL-10 (D), IL-17A (E), IFN-γ (F), and TNF (G) at the indicated stimulation. (H) Heatmaps of percentage of MAIT cell cytokine expression (IL-10, IL-17A, IFN-γ, and TNF) from HC and CD at the indicated stimulation. Lines and error bars represent mean and SE. (B, D–G) Friedman test followed by Dunn’s multiple comparison, *P < 0.05, **P < 0.01, ***P < 0.001, ****P < 0.0001. (H) Wilcoxon test, *P < 0.05, **P < 0.01, ***P < 0.001, ****P < 0.0001.

To evaluate possible differences between health and disease, we directly compared the MAIT cell cytokine production in HC and CD patients. MAIT cell functionality was overall reduced in CD compared to HC with significantly lower levels of IL-10, IL-17A, IFN-γ, TNF, GzmB, and degranulation marker CD107a expression (Fig. 1H and Fig. S1E). To summarize, peripheral blood MAIT cells in CD are reduced in percentage among T cells but retain their ability to shift toward IL-10 production in response to TCR triggering combined with IL-12 stimulus, as well as to produce IL-17A, IFN-γ and TNF following stimulation by 5-OP-RU and IL-18. Despite this, the overall levels of MAIT cell responses to antigen are impaired in CD compared to HC.

### Expression of IL-10 and IL-17A is impaired in MAIT cells in active Crohn’s disease

To further assess if the reduced functionality was driven by the disease state, we compared patients in remission with those with active CD. MAIT cell polyfunctionality was limited in CD compared to HC, independently of disease state and for all stimulatory conditions tested (Fig. 2A). Upon 5-OP-RU and IL-12 stimulation, the co-expression of IL-10, IFN-γ, and GzmB in MAIT cells was decreased in remission and active CD compared to HC. However, the decrease of IL-10^+^GzmB^+^ cells compared to HC was unique to active CD patients (Table 2). IL-10^+^GzmB^+^ MAIT cells were also reduced in the active CD group after stimulation with 5-OP-RU alone (Table 2). In response to 5-OP-RU and IL-18 or IL-12/IL-18 stimulation, MAIT cells expressing the polyfunctional combinations IFN-γ^+^IL-17A^+^TNF^+^GzmB^+^, IFN-γ^+^TNF^+^GzmB^+^, IFN-γ^+^GzmB^+^ were lower in both CD groups compared to HC (Table 2).

**Figure 2:**
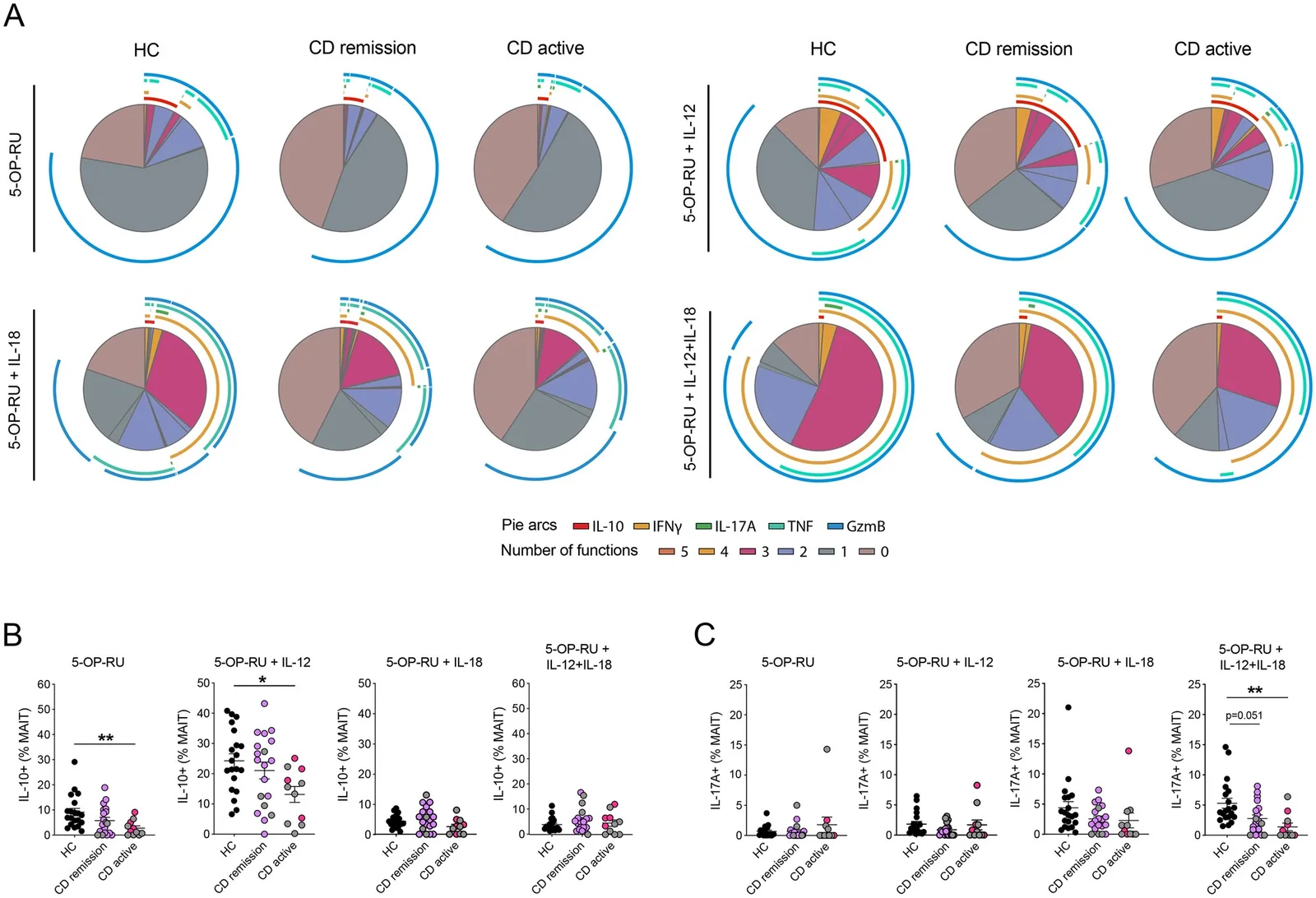
IL-10 and IL-17A production by MAIT cells are impaired in active CD. (A) Pie charts illustrate the polyfunctionality of MAIT cells as determined by intracellular flow cytometry from HC, remission and active CD at the indicated stimulation. Pie arcs indicate the molecules measured (IL-10, IFN-γ, IL-17A, TNF, and GzmB), while pie chart colors represent the number of functions detected (0 to 5). IL-10 (B) and IL-17A (C) expression in MAIT cells in response to the indicated stimulation in HC, remission and active CD. Grey dots indicate patients receiving treatment. Lines and error bars represent mean and SE. Friedman test followed by Dunn’s multiple comparison, *P < 0.05, **P < 0.01.

**Table 2:** Statistical significance of cytokine co-expression in Crohn’s disease patients in remission or active disease versus healthy controls.

	CD remission (*P*-value)	CD active (*P*-value)
**5-OP-RU + IL-12**		
IL-10+ IFNγ+ GzmB+	0.0264	0.0073
IL-10+ GzmB+	NS	0.0039
**5-OP-RU**		
IL-10+ GzmB+	NS	0.002
**5-OP-RU + IL-18**		
IFN-γ+ IL-17A+ TNF+ GzmB+	0.0445	0.0087
IFN-γ+ TNF+ GzmB+	0.0021	0.0032
IFN-γ+ GzmB+	0.0145	0.0176
Non-responsive	0.0017	0.0140
**5-OP-RU + IL-12 + IL-18**		
IFN-γ+ IL-17A+ TNF+ GzmB+	0.0003	0.0001
IFN-γ+ TNF+ GzmB+	0.0007	0.0001
Non-responsive	0.0005	0.0026

CD, Crohn’s disease; NS, Not significant.

At the single-cytokine level, a significant reduction of IL-10 production by MAIT cells was observed only in patients with active CD following stimulation with 5-OP-RU alone or combined with IL-12 (Fig. 2B and Fig. S2A). Furthermore, IL-17A secretion was significantly reduced in active CD in response to 5-OP-RU combined with IL-12 + IL-18 stimulation (Fig. 2C), with a trend toward lower expression in remission. MAIT cell expression of IFN-γ, TNF, GzmB, and CD107a degranulation were all reduced in both remission and active CD compared to HC under different stimulatory conditions, except for TNF where expression in response to 5-OP-RU and IL-12 stimulation was not significantly reduced (Fig. S2B–E). Notably, GzmB expression showed a distinctive pattern as it was more significantly impaired in CD patients in remission in response to 5-OP-RU plus IL-12 or 5-OP-RU plus IL-18 stimulation (Fig. S2E).

Together, these results indicate decreased MAIT cell functionality in both active and remission stage of CD, whereas impairment of in particular IL-10 and to some extend IL-17A is primarily observed in active CD.

### Limited MAIT cell LAG-3 expression in active CD in response to antigen and IL-18

Previous findings indicate that IL-10^+^ MAIT cells specifically upregulate TIM-3, while PD-1 and LAG-3 are broadly increased by activation.^5^ Therefore, we next evaluated inhibitory receptor expression on MAIT cells in CD after stimulation with 5-OP-RU alone or in combination with IL-12 or IL-18, or IL-12 + IL-18. MAIT cells stimulated by 5-OP-RU and IL-12 upregulated TIM-3 expression in HC as well as in active and remission CD patients (Fig. 3A–E). PD-1 and LAG-3 were induced by all stimulation modes and in both states of CD (Fig. 3C and D, Fig. S3A and B). However, the level of LAG-3 expression induced was less in active CD following stimulation with 5-OP-RU alone or in combination with IL-18 (Fig. 3D and F, and Fig. S3C). In contrast, PD-1 expression on MAIT cells showed only a non-significant trend toward reduced induction in CD patients (Fig. 3D and G). Next, the ability of TIM-3^+^, LAG-3^+^, and PD-1^+^ MAIT cells to produce IL-10 upon stimulation with 5-OP-RU and IL-12 was assessed. IL-10 levels were elevated in MAIT cells expressing inhibitory receptors compared to their non-expressing counterparts (Fig. 3H). However, the decreased expression of IL-10 in active disease was only seen in the TIM-3^+^IL-10^+^ MAIT cells (Fig. 3I). Finally, we investigated expression of cytokines by LAG-3^+^ MAIT cells stimulated with 5-OP-RU and IL-18. In active CD, IFN-γ and TNF were reduced in LAG-3^+^ MAIT cells compared to HC (Fig. S3D). At the same time, LAG-3^+^ MAIT cells from HC or CD patients in remission displayed increased IFN-γ and TNF compared to LAG-3^-^ MAIT cells (Fig. S3E). Overall, these findings suggest that patients with active CD upregulate the inhibitory receptor LAG-3 to a lesser extent on MAIT cells in response to stimulation and that the LAG-3^+^ MAIT cells may be less functional.

**Figure 3:**
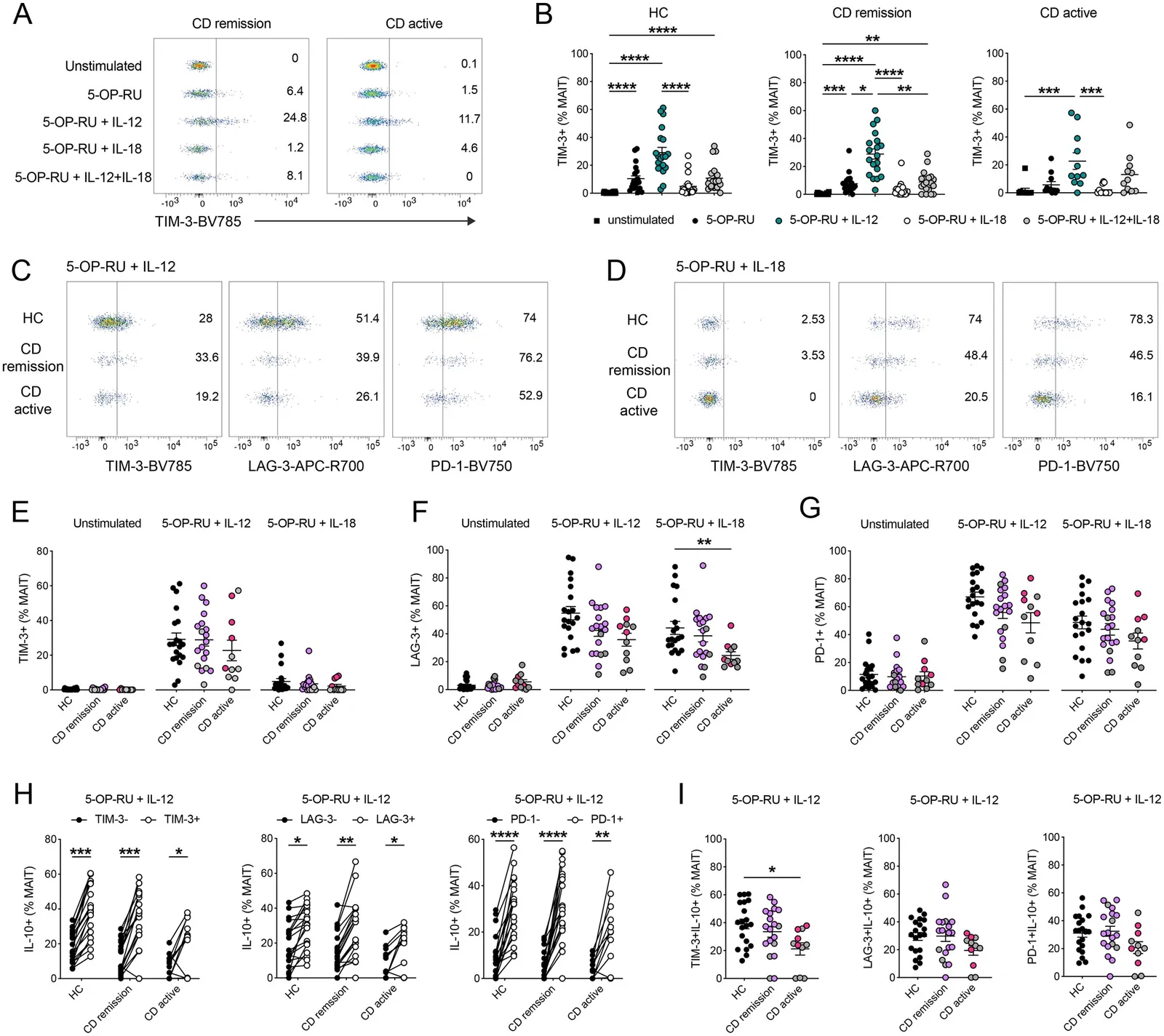
LAG-3 is less induced in MAIT cells in active CD. Representative FACS plots (A) and summary data (B) of TIM-3 expression in MAIT cells at the indicated stimulation in HC, remission and active CD. Representative FACS plots (C-D) and summary data of the expression of TIM-3 (E), LAG-3 (F) and PD-1 (G) on MAIT cells at the indicated stimulation in HC, remission and active CD. (H) Summary data of IL-10 expression by MAIT cells in response to 5-OP-RU and IL-12 stimulation in HC, remission and active CD. (I) Summary data of the co-expression of IL-10 with TIM-3, LAG-3 or PD-1 in MAIT cells stimulated with 5-OP-RU and IL-12 stimulation in HC, remission and active CD. Grey dots indicate patients receiving treatment. Lines and error bars represent mean and SE. (B) Friedman test followed by Dunn’s multiple comparison, *P < 0.05, **P < 0.01, ***P < 0.001, ****P < 0.0001. (E-G, I) Kruskal-Wallis test followed by Dunn’s multiple comparison, *P < 0.05, **P < 0.01. (H) Wilcoxon test, *P < 0.05, **P < 0.01, ***P < 0.001, ****P < 0.0001.

### Transcripts associated with anti-microbial effector function are elevated in MAIT cells in CD ileum

To investigate the quality of MAIT cells at the site of ongoing disease, we leveraged a publicly available scRNAseq data set from sorted CD45^+^CD19^-^CD3^+^ cells of the ileum of 2 CD patients and 2 control patients undergoing surgery for colon cancer or polyp removal.^28^ After quality control, dimensionality reduction, batch correction and clustering, we obtained 7 clusters (Fig. 4A, left). After DGE analysis and selection of genes with adjusted *P*-value <0.05 and log fold change > 1 (Table S3), we identified cluster 0 as resident CD8 T cells (*ITGAE*, *ITGA1*, *ENTPD1*, *KLRD1*, *CD101*) and ɣδ T cells (*TRDC*, *TRGC1*); cluster 1 as activated T cells (*CD69*, *TNAIP3*, *FOS*, *JUN*); cluster 2 as cytotoxic CD8 T cells (*GZMB*, *GZMA*, *GZMM*, *GZMK*, *NKG7*, *IFNG*); cluster 3 as CD4 naive T cells (*CCR7*, *SELL*, *TCF1*, *LEF1*); cluster 4 as T follicular helper cells (*CXCR5*, *BCL6*, *CXCL13*) and Tregs (*FOXP3*, *IL2RA*, *MAF)*; cluster 5 as Type 17 cells (*RORA*, *RORC*, *CXCR6*, *IL17A*); cluster 6 as proliferating T cells (*MKI67*, *PCNA*, *CDK1*, *MCM7*). To identify MAIT cells in UMAP, we used the co-expression of 4 genes: *SLC4A10*, *IL7R*, *KLRB1,* and *CD8A* (Fig. 4A, right). SLC4A10 is highly expressed in MAIT cells.^29–31^ This signature was validated in a scRNAseq data set based on sorted MAIT cells (Fig. S4A).^6^ MAIT cells identified using this signature located on the UMAP primarily on the Type 17 cluster and to some extent on the activated T cell cluster (Fig. 4A).

**Figure 4:**
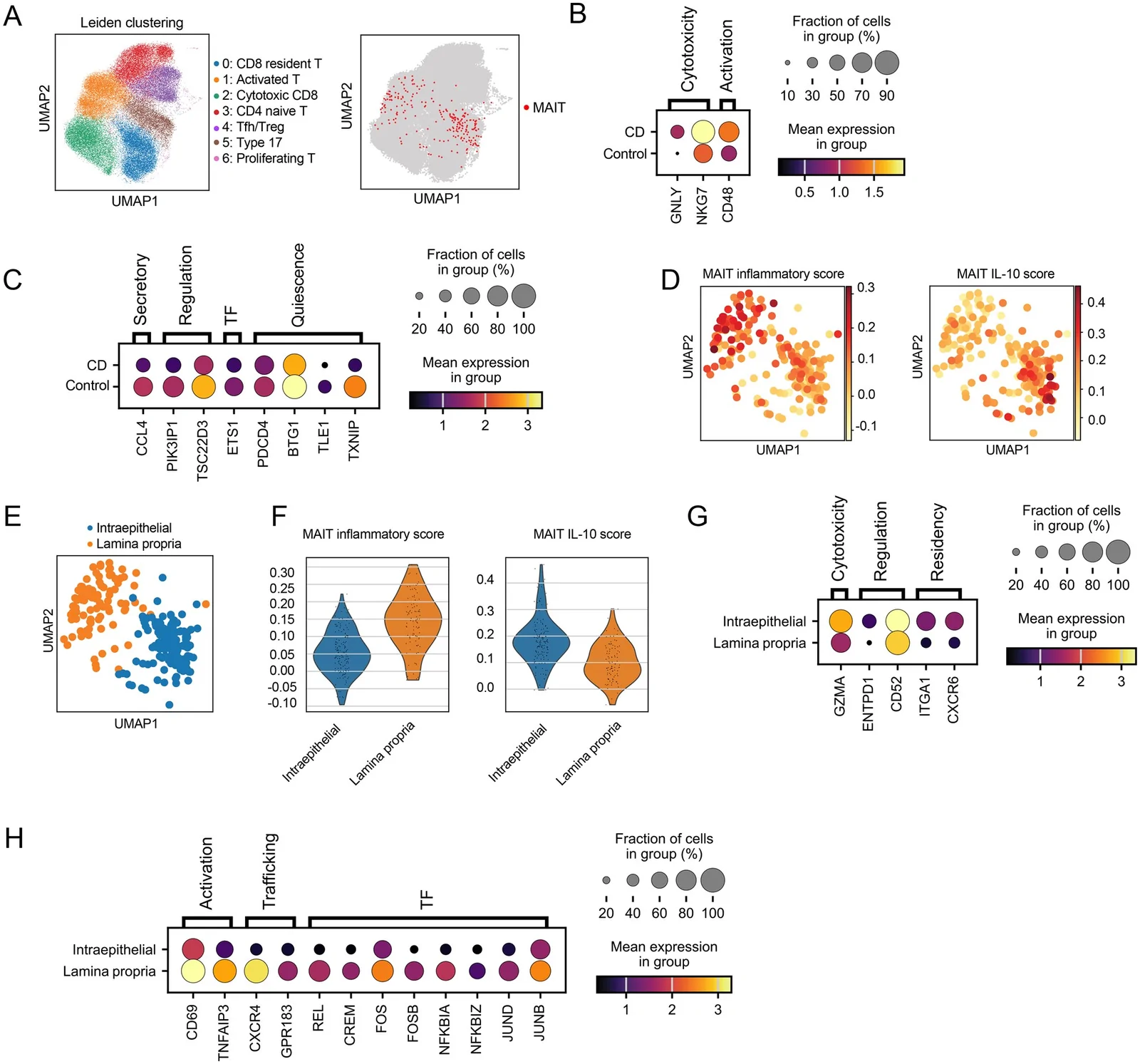
MAIT cells are activated in intestinal CD lesions. (A) UMAP of T cell transcriptome colored by the Leiden clusters at 0.5 resolution (left) or by cells identified as MAIT cells (right). Dot plots of selected genes with log fold change >0.5 and adjusted *P*-values <0.05 in MAIT cells in CD vs control (B) or control vs CD (C). (D) UMAP of sub-clustered MAIT cells displaying MAIT cells inflammatory (left) or IL-10 (right) score. (E) UMAP of sub-clustered MAIT cells with colors showing tissue location. (F) Violin plots of sub-clustered MAIT displaying MAIT cells inflammatory (left) or IL-10 (right) score. (G and H) Dot plots of selected genes with log fold change >0.3 and adjusted *P*-values <0.05 in MAIT cells in CD in the epithelium vs lamina propria (G) or lamina propria vs epithelium (H).

We next performed DGE analysis comparing MAIT cells in CD and control samples, selecting genes with adjusted *P*-value <0.05 and log fold change >0.5. Transcripts associated with anti-microbial effector function (*GNLY*, *NKG7*), and activation (*CD48*) were elevated in MAIT cells from CD patients (Fig. 4B and Table S4). In the healthy controls, transcripts associated with regulation (*PIK3IP1* and *TSC22D3*), quiescence through transcription or cell cycle suppression (*PDCD4*, *BTG1*, *TLE1*, *TXNIP*), and the transcription factor *ETS1* were elevated (Fig. 4C and Table S4).

Expression of *IL10* was low in all cells (Fig. S4B, left) including MAIT cells (Fig. S4B, right). To assess if MAIT cells had regulatory or inflammatory profiles in the tissue, we generated signatures of both the IL-10^+^ regulatory and the IL-18-stimulated inflammatory MAIT cells using our previous scRNAseq data set from peripheral blood MAIT cells (Table S2).^5^ Sub-clustering of MAIT cells indicated that the MAIT cell regulatory and inflammatory signatures localized on distinct regions of the UMAP (Fig. 4D), aligning with the tissue origin of the cells from epithelium or lamina propria, respectively (Fig. 4E; Fig. S4C). Furthermore, the inflammatory signature showed a trend towards higher expression in the lamina propria, whereas the regulatory score exhibited a trend towards enrichment in the epithelium (Fig. 4F). This prompted us to investigate the impact of tissue location on MAIT cell gene expression patterns. DGE analysis of differences between the tissue sites in CD samples (log fold change >0.3, adjusted *P*-value <0.05), indicated that MAIT cells in the intra-epithelial compartment were enriched in transcripts associated with cytotoxicity (*GZMA*), tissue residency (*ITGA1* encoding CD49a and *CXCR6*), regulation (*ENTPD1*, *CD52* that can bind Siglec 10 and induce cell suppression^32^) (Fig. 4G and Table S5). MAIT cells from the lamina propria preferentially expressed genes associated with activation (*CD69*, *TNFAIP3*), trafficking (*CXCR4*, *GPR183* which was shown in mice to be essential to recruit lymphocytes in colitis,^33^) and transcription factors involved in activation (*REL*, *CREM*, *FOS* family, *NFKB* family, *JUND*,) (Fig. 4H and Table S5). In controls, the DGE between lamina propria and epithelial MAIT cells was similar to the pattern in CD for the lamina propria samples only (Fig. S4D and Table S6).

In summary, these findings suggest that intestinal MAIT cells in CD exhibit a transcriptional profile indicative of increased effector functions compared to controls. Furthermore, MAIT cells in the lamina propria and in the epithelium display distinct transcriptional profiles.

### Anti-inflammatory drugs used to treat CD can modulate MAIT cell functions

In our cohort, 10 out of 30 CD patients received treatment with 5-aminosalicylate (5-ASA), corticosteroids or thiopurine (Table 1). Moreover, the JAK inhibitor upadacitinib has been approved for the treatment of CD. To assess the impact of these four drug classes on MAIT cell activation *in vitro*, we stimulated PBMC from healthy donors with 5-OP-RU combined with IL-12 or IL-18 in the presence of either upadacitinib (JAK inhibitor), prednisolone (corticosteroid), mesalazine (5-ASA), azathioprine (thiopurine), or DMSO control. The JAK inhibitor upadacitinib inhibited IFN-γ, TNF, IL-10, IL-22, and GzmB production by MAIT cells irrespective of the stimulation at doses ranging from 0.01 μM to 1 μM (Fig. 5A–D and Fig. S5A, E). However, upadacitinib increased the expression of IL-17A and GM-CSF by MAIT cells upon 5-OP-RU and IL-18 stimulation (Fig. 5D). Prednisolone suppressed MAIT cell activation, markedly inhibiting IFN-γ, TNF, IL-17A, IL-22, GM-CSF, and IL-10 production, as well as reducing GzmB expression (Fig. 5C and D, and Fig. S5A–D). Mesalazine, an anti-inflammatory drug targeting the eicosanoid and prostaglandin pathway,^34^ unexpectedly promoted modestly increased MAIT cell expression of TNF, IL-17A, and IL-22 in response to 5-OP-RU and IL-18 stimulation at doses in the range of 10 μM to 100 μM (Fig. 5B–D and Fig. S5D). Moreover, IFN-γ expression in response to 5-OP-RU and IL-12 stimulation was increased at the dose of 1 μM, below the maximum concentration detected in circulation of treated patients (Fig. 5C). Finally, the thiopurine azathioprine, an immunosuppressant that inhibits purine synthesis and blocks DNA and RNA synthesis,^35^ significantly reduced IFN-γ and TNF expression following 5-OP-RU and IL-12 stimulation (Fig. 5C).

**Figure 5:**
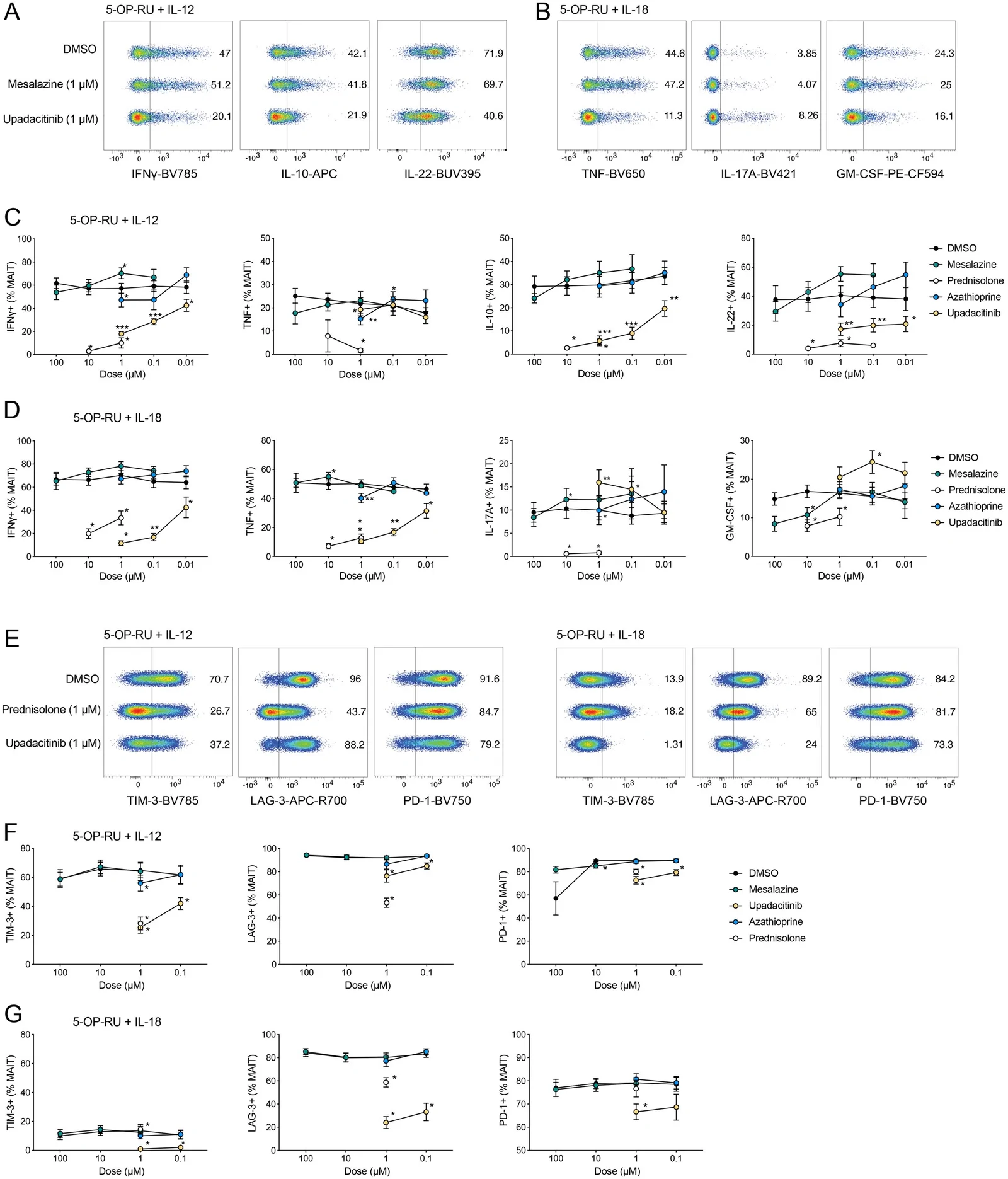
Anti-inflammatory drugs used in CD treatment modulate MAIT cell functions. (A) Representative FACS plots of IFN-γ, IL-10 and IL-22 expression in MAIT cells after stimulation with 5-OP-RU and IL-12 in the presence of either DMSO or mesalazine (1 μM) or upadacitinib (1 μM). (Β) Representative FACS plots of TNF, IL-17A, and GM-CSF expression in MAIT cells after stimulation with 5-OP-RU and IL-18 in the presence of either DMSO control or mesalazine (1 μM) or upadacitinib (1 μM). (C) Summary plots of the frequencies of MAIT cells expressing IFN-γ, TNF, IL-10, and IL-22 after stimulation with 5-OP-RU and IL-12 in the presence of either DMSO or drugs. (D) Summary plots of the frequencies of MAIT cells expressing IFN-γ, TNF, IL-17A, and GM-CSF after stimulation with 5-OP-RU and IL-18 in the presence of DMSO or drugs. Representative FACS plots (E) and summary data (F, G) of ΤΙΜ-3, LAG-3 and PD-1 expression on MAIT cells at the indicated stimulation in the presence of drugs or DMSO at the indicated concentration. Wilcoxon test, *P < 0.05, **P < 0.01, ***P < 0.001.

Next, we investigated whether these drugs affected the expression of the inhibitory receptors TIM-3, PD-1, and LAG-3 on MAIT cells. Prednisolone and upadacitinib inhibited the upregulation TIM-3, PD-1, and LAG-3 on the cell surface of stimulated MAIT cells (Fig. 5E, F, and G). However, the expression of these inhibitory receptors remained stable in the presence of mesalazine and azathioprine (Fig. 5F and G).

To assess whether these treatments affected MAIT cells in vivo, we analyzed the cytokines and inhibitory receptors expression on MAIT cells of treated and non-treated CD patients. Due to small sample size in each treatment group, no significant effect could be observed between treated and untreated patients (Fig. S5F–I). However, corticosteroid treatment trended towards the lowest functional MAIT cell response while patients treated with 5-ASA and azathioprine had either a decreased functionality or a response comparable to untreated patients (Fig. S5F–I).

In summary, the corticosteroid prednisolone suppressed MAIT cell activation. The JAK inhibitor upadacitinib reduced MAIT cell activation and induction of inhibitory receptors but supported an increase of IL-17A and GM-CSF following 5-OP-RU and IL-18 stimulation. Mesalazine and azathioprine exerted only mild effects on MAIT cell functions.

## Discussion

MAIT cells are unconventional T cells that recognize riboflavin pathway-derived antigens from many microbial species, and their functional plasticity enables their responses to be shaped by the surrounding tissue microenvironment. CD is an inflammatory condition characterized by an increased abundance of pro-inflammatory cytokines, such as IL-18, TNF and IL-23. At the same time, increased expression of IL-10 by immune cells has been reported but seems insufficient to balance the inflammation.^36^ Targeting pro-inflammatory cytokines is a treatment strategy in IBD. Blockade of IL-18 may improve colitis in animal models,^37^ but has not yet been tested clinically. Anti-TNF therapy is available for both CD and ulcerative colitis^36^ and the efficacy depends on increased production of IL-10 by macrophages.^38^ Antibodies directed against IL-23 seem to help only subgroups of patients.^36^ The potential influence of the inflammatory microenvironment and the effect of various treatments on MAIT cells in CD patients have not been thoroughly investigated. Here, we found that peripheral blood MAIT cells from CD patients adjust their functional response profile to the cytokine microenvironment, similar to MAIT cells from healthy donors. However, MAIT cells in both remission and active stages of CD displayed overall reduced effector functions in terms of IFN-γ and TNF expression, and reduced GzmB expression and CD107a degranulation. In the inflammatory context of CD, where enhanced levels of MAIT cell antigen are found in the circulation,^21^ MAIT cells might contribute to the on-going inflammation with release of IFN-γ and TNF. However, the lower ability of MAIT cells to produce those cytokines in CD compared to HC may be a consequence of chronic activation.

We find that MAIT cells have preserved capacity to express IL-10 and IL-17A in CD remission phase, but expression of these cytokines was impaired during active disease. IL-17A is generally increased in IBD but blocking IL-17A with monoclonal antibodies in patients with CD often worsens the disease,^39–41^ possibly due to the role of IL-17A in maintaining intestinal barrier integrity by promoting tight junctions and repair mechanisms in the epithelium.^36^^,^^42–44^ Expression of the inhibitory receptor LAG-3 was preserved on MAIT cells during CD remission phase but was reduced in active disease upon inflammatory stimulation. Interestingly, LAG-3 was previously found to be essential for Treg-mediated suppression of the CX3CR1^+^ macrophages that drive inflammation in a mouse model of induced colitis.^45^ The reduced expression of IL-17A, IL-10, and LAG-3 in MAIT cells in active CD may potentially limit their role in barrier maintenance and regulatory functions during active stages of disease. However, the upregulation of TIM-3 and PD-1 on MAIT cells in response to stimulation was maintained in CD. The role for TIM-3 in IBD is supported by results in a murine model of colitis, where TIM-3 blocking worsened the disease, through the enhancement of Th1 and Th17 responses and the reduction of regulatory T cells.^46^ Thus, the recovery of IL-10 and IL-17A during the remission phase of the disease combined with the maintenance of TIM-3 and PD-1 on MAIT cells upon stimulation, suggests that MAIT cell could potentially perform at least some of their regulatory and barrier functions when the symptoms are under control.

Intestinal barrier integrity is defective in CD, and this plays a role in pathogenesis. The higher permeability of the intestine allows microbial translocation and leakage of antigens into the tissues.^47^ Studies in mice have indicated that MAIT cells are involved in intestinal barrier maintenance. Mice deficient in MAIT cells have impaired barrier integrity,^23^^,^^48^ and MAIT cells can perform tissue repair functions in the intestine.^10^ Furthermore, IL-10, IL-17A, and IL-22, which are produced by MAIT cells,^5^^,^^6^^,^^49^ are involved in preserving barrier integrity.^47^ In the present study, we found that intestinal MAIT cells in CD displayed a transcriptional profile pattern with elevated *GNLY* and *NKG7*, while MAIT cells from controls expressed genes related to quiescence. In previous studies of IBD, human MAIT cells were found to be recruited to the gut.^17^^,^^19^ It is possible that a fraction of intestinal MAIT cells in CD could be newly recruited cells from the circulation to promote a local inflammatory process rather than barrier maintenance.

MAIT cells are found in both the lamina propria and the epithelium of the intestine, although their abundance differs between the two sites.^50^ We found that MAIT cells in the epithelium display a tissue residency program associated with a capacity for regulation and cytotoxicity, while lamina propria MAIT cells display transcripts associated with activation and recruitment to tissue sites. It is tempting to speculate that MAIT cells in the lamina propria of CD patients may be newly recruited cells from the periphery, while the intra-epithelial tissue-resident population may still perform some regulatory function. At steady-state, human tissue-resident MAIT cells in the intestines have a regulatory profile with CD39 expression and IL-10 production.^6^ Interestingly, another unconventional innate-like T cell population, the γδ T cells, promotes tolerance in the intestinal epithelium in a murine model of ileitis.^51^ Furthermore, dichotomy in transcriptional profiles between lamina propria and epithelium has been described for conventional tissue resident memory CD8 T cells.^52^ It is important to note that our scRNAseq analysis is limited by the small sample size and should be viewed as hypothesis generating. The analysis is also limited by the transcriptional definition of intestinal MAIT cells, which was based on a validated signature of peripheral blood MAIT cells. While this approach will accurately identify MAIT cells, it is possible that cells with atypical tissue-dependent signatures may be missed. Future studies should therefore be designed to better define the diversity of transcriptional signatures of intestinal MAIT cells identified by MR1 tetramer-based sorting. Altogether, renewed and larger transcriptional studies of MAIT cells in CD will be important to validate these findings.

A range of anti-inflammatory drugs are currently used to treat IBD but their impact on MAIT cell function remains largely unclear. Furthermore, MAIT cells express the multidrug resistance transporter ABCB1 that confers resistance to some chemotherapies and xenobiotics.^7^ The thiopurine azathioprine, which is a chemotherapy agent, was previously found not to affect MAIT cell numbers in IBD.^53^ Consistent with this, we found that thiopurine and 5-ASAs only partly inhibited MAIT activation in response to stimulation, suggesting that both drug classes may not act directly on MAIT cells or could possibly be exported by the ABCB1 transporter. Prednisolone, a corticosteroid drug, directly dampened MAIT cell activation, consistent with findings by Sattler et al.,^54^ limiting both cytokine production and the upregulation of inhibitory receptors. The JAK inhibitor upadacitinib, which has higher specificity for JAK1 than JAK2,^55^ inhibited the MAIT cell response to 5-OP-RU and IL-12 or IL-18, although IL-12R relies on JAK2 and IL-18R depends on the MAP kinase pathway for signaling. Interestingly, upadacitinib promoted increased IL-17A and GM-CSF expression by MAIT cells when stimulated with 5-OP-RU in combination with IL-18. In mice, JAK inhibitor treatment increases IL-17A production by CD4 T cells in experimental autoimmune encephalitis,^56^ whereas these drugs were shown to decrease Th17 responses in vitro.^57^ Furthermore, the JAK inhibitor ruxolitinib was found to re-invigorate exhausted T cells with increased GM-CSF secretion in vivo, including in proliferating T cells.^58^ The precise mechanism of how JAK inhibitors modulate Th17 immunity in different microenvironments remains to be investigated. Our cohort had too few treated patients to validate the *in vitro* findings. However, we speculate that MAIT cell function may not be affected in CD patients receiving azathioprine and mesalazine, while patients receiving corticosteroids may have dampened MAIT cell responses. The increased IL-17A and GM-CSF expression observed in MAIT cells in response to inflammatory stimulus in the presence of upadacitinib warrants further investigation.

In summary, our findings indicate that the MAIT cell compartment is compromised in CD, as evidenced by reduced numbers and reduced expression of key inflammatory cytokines and cytolytic effector proteins. At the same time, regulatory and barrier maintenance functions expressed by circulating MAIT cells were significantly impaired during the active phase of CD but seemed to recover during the remission phase. At lesional sites MAIT cells display a profile in line with the notion that they participate in the inflammatory process. Moreover, distinct signatures according to the localization suggest a more regulatory role for MAIT cells in the epithelium and a more activated phenotype in the lamina propria. Finally, conventional anti-inflammatory drugs currently used to treat CD modify MAIT cell activation. This study provides new insights into the complex roles of MAIT cells in CD.

## Supplementary Material

vkag271_Supplementary_Data

## Data Availability

The authors declare that the data supporting the findings of this study are available within the article and its supplementary information files or are available upon reasonable requests to the authors.

## Abbreviations

CD: Crohn’s disease
DGE: differential gene expression
GzmB: granzyme B
HC: healthy controls
IBD: inflammatory bowel disease
MAIT: mucosa-associated invariant T
scRNAseq: single cell RNA-sequencing, 5-ASA, 5-aminosalicylate
5-OP-RU: 5-(2-oxopropylideneamino)-6-D-ribitylaminouracil
