# Supplementary material for: Altered MAIT cell activation characteristics with impaired IL-10 and IL-17A response programs in active Crohn’s disease
Source: J Immunol. 2026 Oct 26;215(10):vkag271. doi: 10.1093/jimmun/vkag271 (PMC13707261; doi:10.1093/jimmun/vkag271)
Supplement: Supplementary file 1 [file vkag271_supplementary_data.zip › Mouchtaridi CD Supplementary Figures and tables.pdf]

Supplementary material for the paper:

Altered MAIT cell activation characteristics with impaired IL-10 and IL-17A response programs in active Crohn's disease

Elli Mouchtaridi[1], Jonas Halfvarson[2], Jeffrey Y. W. Mak[3], David P. Fairlie[3], Jenny Mjösberg[1,4], Peter Bergman[5,6], Johan K. Sandberg[1,*], Caroline Boulouis[1,*]

[1]Center for Infectious Medicine, Department of Medicine, Karolinska Institutet, Stockholm, Sweden. [2]Department of Gastroenterology, Faculty of Medicine and Health, Örebro University, Örebro, Sweden. [3]Centre for Chemistry and Drug Discovery, Australian Research Council Centre of Excellence for Innovations in Peptide and Protein Science, Institute for Molecular Bioscience, The University of Queensland, Brisbane, Australia. [4]Clinical Lung and Allergy Research Unit, Medical Unit for Lung and Allergy Diseases, Karolinska University Hospital Huddinge, Stockholm, Sweden. [5]Department of Laboratory Medicine, Division of Clinical Immunology, Karolinska Institutet, Stockholm, Sweden. [6]Department of Clinical Immunology and Transfusion Medicine, Karolinska University Hospital, Stockholm, Sweden.

***Correspondence to:** Dr. Caroline Boulouis, Center for Infection Medicine, Department of Medicine, Karolinska Institutet, Alfred Nobels Allé 8, 14152 Stockholm, Sweden. E-mail: caroline.boulouis@ki.se

or to Dr. Johan K. Sandberg, Center for Infection Medicine, Department of Medicine, Karolinska Institutet, Alfred Nobels Allé 8, 14152 Stockholm, Sweden. E-mail: johan.sandberg@ki.se

This document contains 5 supplementary figures and 1 supplementary table

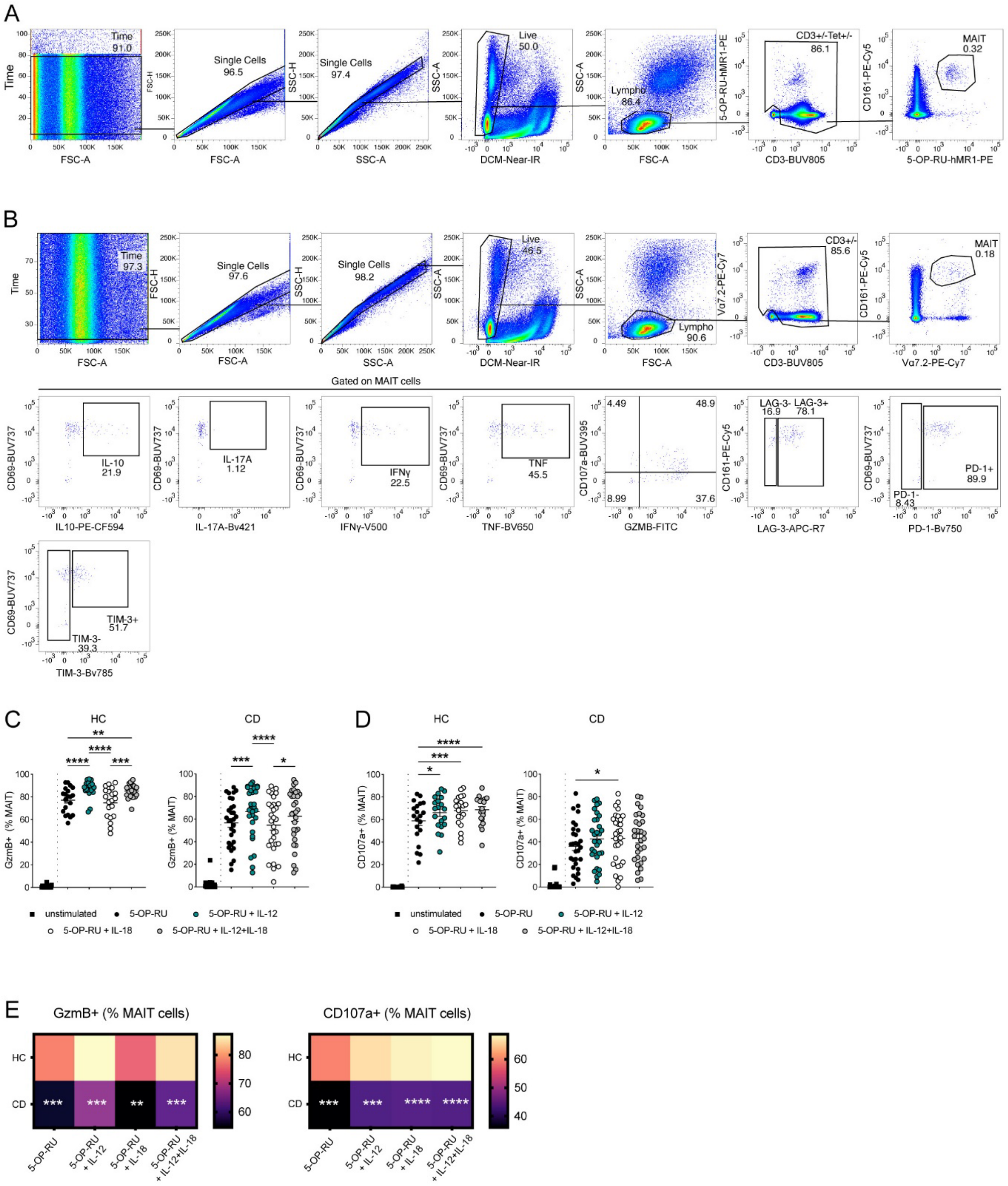

Supplementary Figure 1. (A) Gating strategy for identification of MAIT cells in PBMC. (B) Gating strategy for identification of MAIT cells after stimulation and example plots of surface and intracellular staining in stimulated cells. Summary data of GzmB production (C) and CD107a degranulation (D) in MAIT cells at the indicated stimulation in HC, remission and active CD. (E) Heatmaps of expression percentage of GzmB and CD107a by MAIT cells in HC and CD at the indicated stimulation. (C, D) Friedman test followed by Dunn's multiple comparison, *p<0.05, **p<0.01, ***p<0.001. Lines and error bars represent mean and SE. (E) Wilcoxon test, *p<0.05, **p<0.01, ***p<0.001.

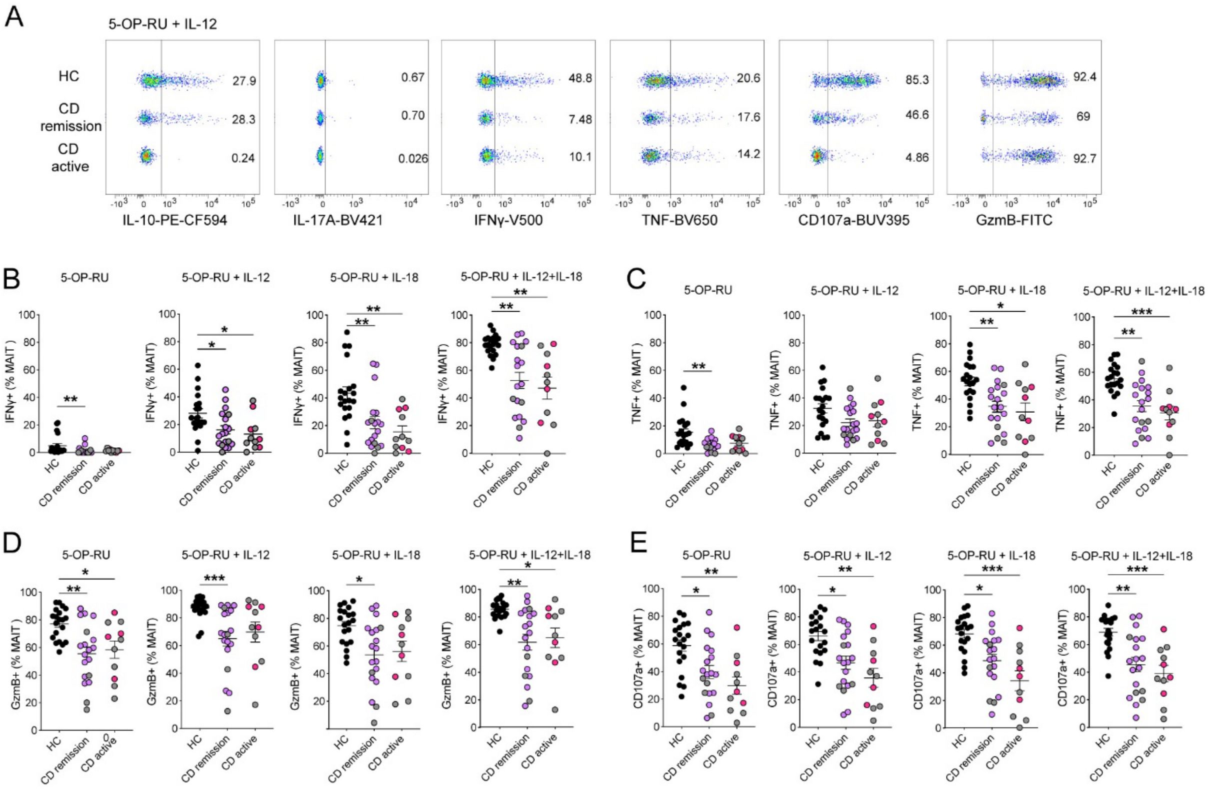

Supplementary Figure 2. MAIT cell functions are reduced in remission and active CD. (A) Representative FACS plots of IL-10, IL-17A, IFNγ, TNF, GzmB, and CD107a in MAIT cells after stimulation with 5-OP-RU and IL-12 in HC, remission and active CD. Summary data of IFNγ (B), TNF (C), GzmB (D) and CD107a (E) frequencies of MAIT cells at the indicated stimulation in HC, remission and active CD patients. Grey dots indicate patients receiving treatment. Lines and error bars represent mean and SE. Friedman test followed by Dunn's multiple comparison, *p<0.05, **p<0.01, ***p<0.001.

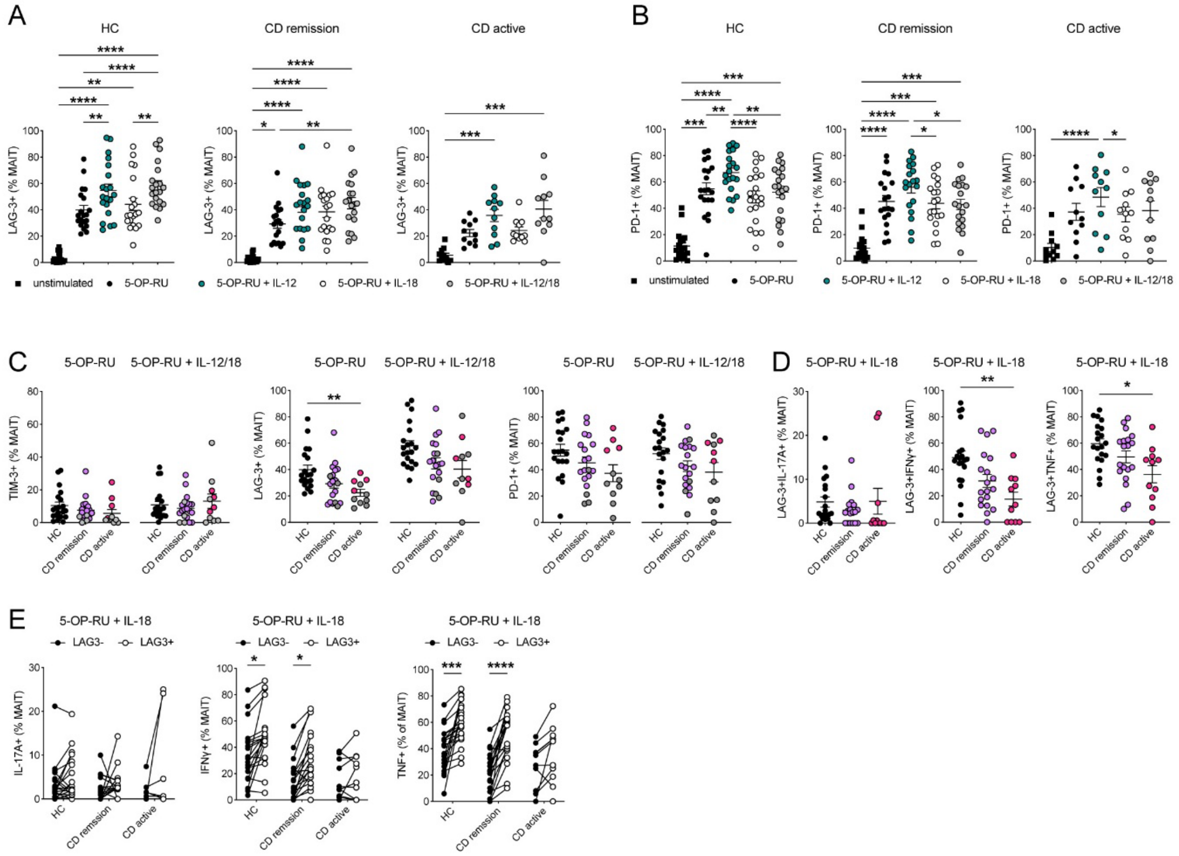

Supplementary Figure 3. LAG-3 and PD-1 are shifted by the cytokine environment in CD. Summary data of LAG-3 (A) and PD-1 (B) expression by MAIT cells at the indicated stimulation in HC, remission and active CD. (C) Summary data of TIM-3, LAG-3, and PD1 expression by MAIT cells at the indicated stimulation in HC, remission and active CD. (D, E) Co-expression of LAG-3 with the indicated cytokines after 5-OP-RU and IL-18 stimulation. Grey dots indicate patients receiving treatment. Lines and error bars represent mean and SE. (A, B) Friedman test followed by Dunn's multiple comparison. *p<0.05, **p<0.01, ***p<0.001, ****p<0.0001. (C) Kruskal Wallis test followed by Dunn's multiple comparison, **p<0.01.

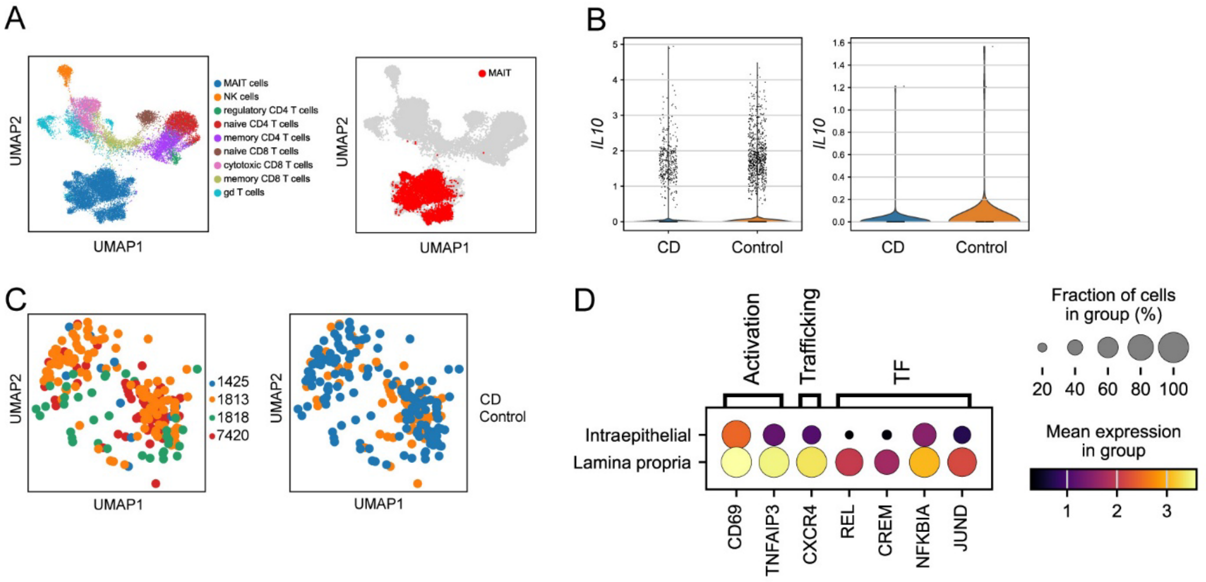

Supplementary Figure 4. (A) UMAP colored by cell type identified in Kamman et al. (left) or identified as MAIT cells in this study (right). (B) Violin plots of *IL10* expression in the whole dataset (left) or in MAIT cells (right). (C) UMAP of sub-clustered MAIT cells with colors showing donors (left), or disease state (middle). (D) Dot plots of selected genes with log fold change >0.3 and adjusted p-values <0.05 in MAIT cells in control in the epithelium vs lamina propria (left) or lamina propria vs epithelium (right).

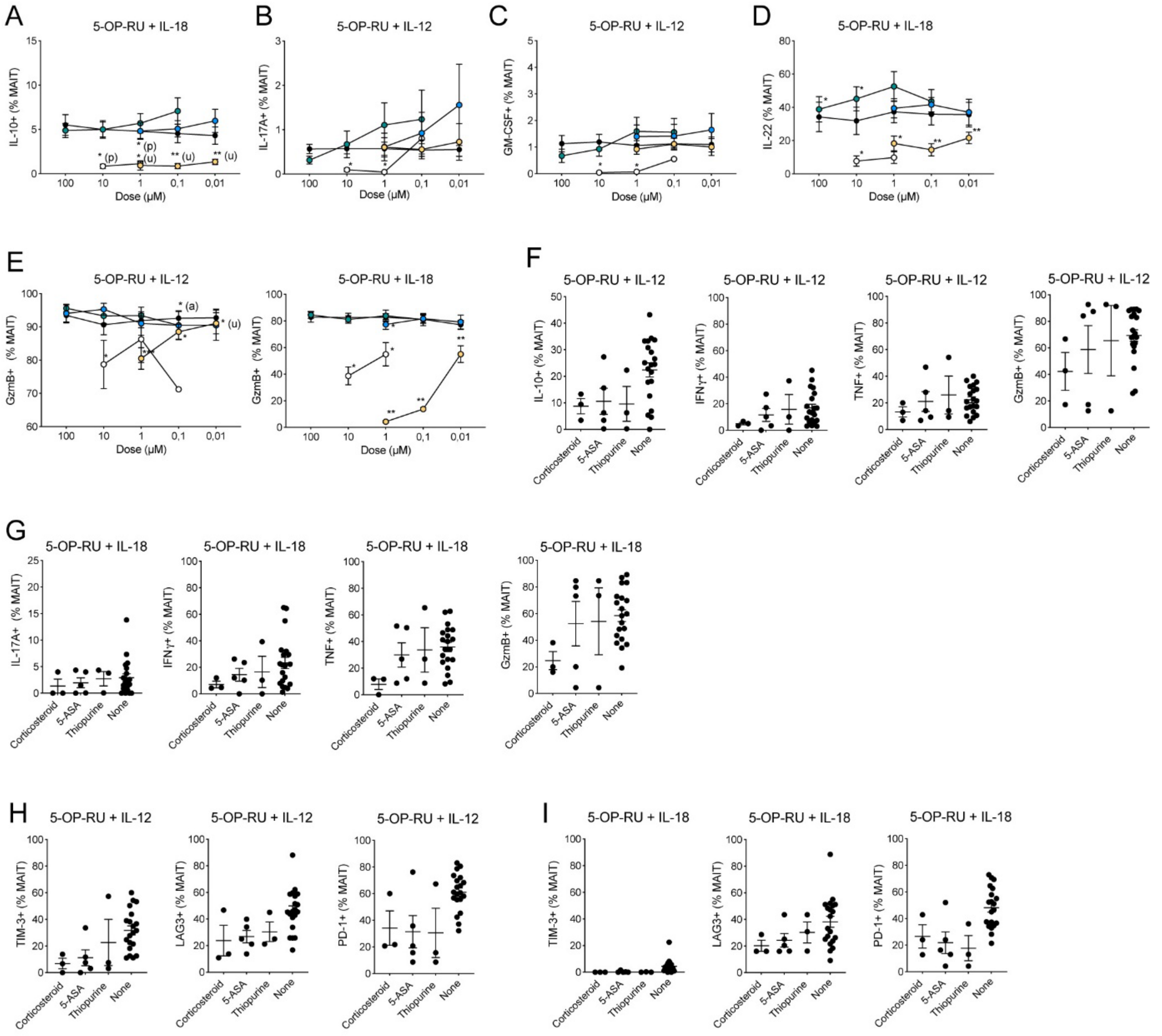

Supplementary Figure 5. Anti-inflammatory drugs effect on MAIT cell functionality. Summary plots of the frequencies of MAIT cells producing IL-10 (A), IL-17A (B), GM-CSF (C), IL-22 (D) and GzmB (E) at the indicated stimulation in the presence of DMSO or drugs at the indicated concentration. Summary data of IL-10 or IL17A and IFNγ, TNF and GzmB expression in MAIT cells from CD patients stimulated with 5-OP-RU and IL-12 (F) or 5-OP-RU and IL-18 (G). Summary data of TIM-3, LAG-3 and PD-1 expression in MAIT cells from CD patients stimulated with 5-OP-RU and IL-12 (H) or 5-OP-RU and IL-18 (I). Wilcoxon test, *p<0.05, **p<0.01.

Table S1. Monoclonal antibodies and reagents used in flow cytometry.

Antigen	Fluorochrome	Clone	Provider
Vα7.2<br>CD3<br>CD8<br>CD4<br>TIM-3<br>PD-1<br>LAG-3<br>IL-17A<br>IFNɣ<br>GzmB	PE-Cy7<br>BUV805<br>Bv570<br>Bv711<br>Bv785<br>Bv750<br>AF647<br>Bv421<br>Bv785<br>FITC	3C10<br>SK7<br>RPA-T8<br>OKT4<br>F38-2E2<br>EH12.2H7<br>11C3C65<br>BL168<br>4SB3<br>GB11	BioLegend
CD161<br>IL-10<br>IL-10<br>CD69<br>LAG-3<br>CD163<br>IFNɣ<br>TNF<br>GM-CSF<br>GzmB<br>CD107a<br>IL-22	PE-Cy5<br>PE-CF594<br>APC<br>BUV737<br>APC-R700<br>PE<br>V500<br>Bv650<br>PE-CF594<br>R718<br>BUV395<br>BUV395	DX10<br>JES3-19F1<br>JES3-19F1<br>FN50<br>T47-530<br>GHI/61<br>B27<br>Mab11<br>BVD2-21C11<br>GB11<br>H4A3<br>MH22B2	BD Biosciences
Live/Dead Near IR			Invitrogen
5-OP-RU-hMR1	PE	N/A	NIH Tetramer Core Facility
